# Lung Cancer Attributed Mortality Among 316,336 Early Stage Breast Cancer Cases Treated by Radiotherapy and/or Chemotherapy, 2000–2015: Evidence From the SEER Database

**DOI:** 10.3389/fonc.2020.602397

**Published:** 2021-02-25

**Authors:** Semaw Ferede Abera, Rafael T. Mikolajczyk, Eva Johanna Kantelhardt, Ljupcho Efremov, Ahmed Bedir, Christian Ostheimer, André Glowka, Dirk Vordermark, Daniel Medenwald

**Affiliations:** ^1^ Department of Radiation Oncology, Faculty of Medicine, Martin-Luther-University Halle-Wittenberg, Halle (Saale), Germany; ^2^ Institute for Medical Epidemiology, Biometrics and Informatics (IMEBI), Interdisciplinary Center for Health Sciences, Medical School of the Martin-Luther-University Halle-Wittenberg, Halle (Saale), Germany; ^3^ Department of Gynaecology, Faculty of Medicine, Martin-Luther-University Halle-Wittenberg, Halle (Saale), Germany

**Keywords:** radiotherapy, lung cancer, breast cancer, general population, risk, chemotherapy

## Abstract

**Objective:**

To estimate the risk of death from lung cancer in patients treated for breast cancer (BC) in relation to the general population.

**Methods:**

BC data, covering 2000 to 2015, were extracted from the Surveillance, Epidemiology and End Results-18 (SEER-18) cancer registry database. A comparison of lung cancer attributed mortality between BC patients and the general population was performed using standardized mortality ratios (SMRs) and SMRs conditional on survival length (cSMRs). Prognostic factors of lung cancer mortality were identified using flexible parametric modelling. Our model adjusts the effect of downstream (histopathological BC tumor grade and hormone receptor status) and upstream (age at diagnosis, ethnicity, and marital status) factors.

**Results:**

The median follow-up was 6.4 years (interquartile range, 3.0–10.3 years). BC cases who received only radiotherapy (cSMR = 0.93; 95%CI: 0.77–1.13), only chemotherapy (cSMR = 0.91; 0.62–1.33), and radio-and chemotherapy (cSMR = 1.04; 0.77–1.39) had no evidence of increased lung cancer mortality relative to the general population. The adjusted model identified that lung cancer mortality was higher for women who were older at diagnosis compared to those <50 years (ranging from HR50-59 = 3.41 [95%CI: 2.72–4.28] to HR70-79 = 10.53 [95%CI: 8.44–13.13]) and for cases with negative estrogen and progesterone receptors (HR =1.38; 95% CI: 1.21–1.57). Compared to married cases, widowed, divorced, single or others had a 76%, 45%, and 25% higher hazard of lung cancer mortality, respectively. Lung cancer mortality was lower for American Indian/Alaska Native and Asian/Pacific Islander ethnicities (HR = 0.51; 95% CI: 0.40–0.64) compared to BC cases with white ethnic background.

**Conclusions:**

There is no evidence for a higher lung cancer mortality in BC patients when compared to the general population.

## Introduction

On a global scale, female breast cancer (BC) is one of the cancer types with the highest incidence and a leading cause of death (CoD) with age-standardized incidence and mortality rates of 45.3 and 15.9 per 100,000 population in 2017, respectively (1, 2). In the United States, more than 3.8 million women were reported to live with history of invasive BC in 2019 and the life-time risk of having BC was 12.4% (3, 4).

Due to the advent of early screening and multidisciplinary treatment (5–8), the health loss from BC is currently decreasing, although there is still need for an optimization of cancer care (9, 10). These improvements in BC health services have led to increased survival among BC patients (11). However, deaths from secondary cancers after BC diagnosis could negatively impact long-term survival (12). Studies have reported that radiotherapy for BC has been linked to increased risk of lung cancer incidence and mortality (12–17). Analyses based on SEER data addressing ipsilateral versus contralateral lung cancer mortality indicated that the risk elevation started 10 years after radiotherapy and increased over time since the initial radiotherapy (14, 16). In a similar line, a meta-analysis of cohort studies analysing secondary non-breast cancers, indicated a peak between 10 and 15 years after breast cancer diagnosis (17). Other studies suggested that modern radiotherapy methods (3D planning) are associated with a lower mortality risk than earlier treatments (12, 14). Based on data from randomized trials, Taylor et al. estimated the excess risk ratio (ERR) for incident lung cancer and subsequently lung cancer mortality per Gy (15). They compute an ERR of 0.11 per Gy whole-lung dose 10 years after therapy. Implications depended strongest on the additional risk related to smoking.

Using recent data with a sufficiently long follow-up, we analyzed the mortality ratio compared to general population.

In addition, we investigated the down- (clinico-pathological) and upstream (socio-demographic) prognostic factors for lung cancer-attributed mortality and the competing CoD among BC patients.

## Methods and Materials

### Data Sources and BC Cases

The National Cancer Institute (NCI) through its Surveillance, Epidemiology and End Results-18 (SEER-18) program collects detailed population-based cancer data (18). This data platform, which includes 18 cancer registries covering nearly 30% of the US population, is considered to be representative in terms of poverty and education measures (18). Information on CoDs is traced by the assigned codes using the 10^th^ International Classification of Diseases (ICD-10) (19). Our study uses the SEER BC data collected between January 1, 2000 and December 31, 2015 for BC cases aged 15 years or older. Data on the US female population, along with the crude lung cancer and all-cause mortality rates stratified by age and calendar period, were obtained from the Wide-ranging ONline Data for Epidemiologic Research of the Centers for Disease Control and prevention (CDC WONDER), an information management architecture designed to provide scientific public health information (20, 21).

All cases with early stage BC (T1-T3N_0_), known history of surgery, known BC stage (excluding stage 0) and recorded ICD-10 codes (for both CoD and BC diagnosis) were included in this study. Since triple negative BC cases have a high risk of BC metastases, these cases were also excluded. For the purpose of further analyses, the inclusion criteria were narrowed to retain only those who were treated by radio-or chemotherapy or both (irrespective of endocrine and/or targeted treatment). We performed treatment specific analyses in order to account for different indications for radio and/or chemotherapy according to TNM-stage, human epidermal growth factor receptor 2 (HER2), hormone receptor and lymph node status according to NCCN guidelines (https://www.nccn.org/). In general, after breast conserving surgery radiotherapy is part of the general treatment recommendation with chemotherapy being applied according to tumor size, nodal status, hormone receptor status, and gene assay (Oncotype DX Breast Cancer Recurrence Score^®^). Finally, we identified 316,336 eligible patients (**Supplementary Figure 1**). To compare how the treated patients differ from the patients for whom treatment was not documented in SEER, information for both groups (n = 522,982 patients) is summarized in **Table 1**.

**Table 1:** Baseline characteristics by breast cancer treatment history in the United States, 2000–2015 (n = 522,982 cases).

Characteristics	Categories	Treatment (Radiotherapy or chemotherapy or both), row number (%)		Total (Column %)
		Yes	No or unknown^a^	
Total		316,336 (60.5)	206,646 (39.5)	522,982
Age at diagnosis	<50	68,705 (70.3)	28,961 (29.7)	97,666 (18.7)
	50–59	84,633 (68.9)	38,138 (31.1)	122,771 (23.5)
	60–69	89,031 (65.6)	46,710 (34.4)	135,741 (25.9)
	70–79	56,289 (52.4)	51,203 (47.6)	107,492 (20.6)
	≥80	17,678 (29.8)	41,634 (70.2)	59,312 (11.3)
Age at diagnosis, mean±SD		59.9±12.3	66.4 ± 14.0	62.5±13.4
Median survival (IQR) in years		6.4 (3.0–10.3)	5.4 (2.4–9.2)	6.0 (2.7–9.9)
Ethnicity	White	262,170 (60.3)	172,606 (39.7)	434,776 (83.1)
	Black	27,402 (62.2)	16,656 (37.8)	44,058 (8.4)
	Others	26,764 (60.6)	17,384 (39.4)	44,148 (8.5)
Marital status	Married	189,780 (64.9)	102,752 (35.1)	292,532 (55.9)
	Single	39,122 (61.8)	24,222 (38.2)	63,344 (12.1)
	Divorced	34,051 (63.6)	19,514 (36.4)	53,565 (10.2)
	Widowed	38,757 (44.8)	47,702 (55.2)	86,459 (16.5)
	Separated/unknown status/others	14,626 (54.0)	12,456 (46.0)	27,082 (5.2)
Year of diagnosis	2000–2005	109,283 (59.7)	73,623 (40.3)	182,906 (35.0)
	2016–2010	102,119 (61.4)	64,240 (38.6)	166,359 (31.8)
	2011–2015	104,934 (60.4)	68,783 (39.6)	173,717 (33.2)
Stage	I	239,837 (60.1)	159,358 (39.9)	399,195 (76.3)
	II	76,499 (61.8)	47,288 (38.2)	123,787 (23.7)
Histopathological tumor grade	Low	81,767 (57.5)	60,342 (42.5)	142,109 (27.2)
	Moderate	130,730 (59.3)	89,528 (40.7)	220,258 (42.1)
	High	89,314 (68.2)	41,610 (31.8)	130,924 (25.0)
	Unknown/NA	14,525 (48.9)	15,166 (51.1)	29,691 (5.7)
Deceased	Yes	42,899 (44.5)	53,417 (55.5)	96,316 (18.4)
	No	273,437 (64.1)	153,229 (35.9)	426,666 (81.6)
Cause of death	Alive	273,437 (64.1)	153,229 (35.9)	426,666 (81.6)
	Lung cancer	2,276 (50.5)	2,227 (49.5)	4,503 (0.9)
	Breast cancer	12,894 (55.3)	10,444 (44.6)	23,338 (4.4)
	All-ther causes	27,729 (40.5)	40,746 (59.5)	68,475 (13.1)
Radiotherapy	Yes	256,002 (100)	0 (0)	256,002 (48.9)
	No/Unknown	60,334 (22.6)	206,646 (77.4)	266,980 (51.1)
Chemotherapy	Yes	127,508 (100)	0 (0)	127,508 (24.4)
	No/Unknown	188,828 (47.7)	206,646 (52.3)	395,474 (75.6)
HER2^b^	Positive	19,970 (76.0)	6,291 (24.0)	26,261 (12.8)
	Negative	97,506 (59.3)	66,941 (40.7)	164,447 (80.2)
	Bordeline/unknown	6,402 (44.4)	8,014 (55.6)	14,416 (7.0)
ER	Positive	255,778 (60.7)	165,797 (39.3)	421,575 (80.6)
	Negative	46,964 (70.8)	19,369 (29.2)	66,333 (12.7)
	Bordeline/unknown	13,594 (38.8)	21,480 (61.2)	35,074 (6.7)
PR	Positive	218,864 (60.7)	141,579 (39.3)	360,443 (68.9)
	Negative	80,269 (66.4)	40,691 (33.6)	120,960 (23.1)
	Bordeline/unknown	17,203 (41.4)	24,376 (58.6)	41,579 (8.0)

^a^206, 646 (39.5%) cases did not receive treatment or their treatment status is unknown and are excluded from the study. ^b^Not available for 317,858 (60.8%) cases since HER2 status is recorded in SEER since 2010+ onwards. IQR, Interquartile range; NA, Not applicable; HER2, Human epidermal growth factor receptor 2; ER, Estrogen receptor; PR, Progesterone receptor.

### Follow-Up, Measurement, and Outcome Ascertainment

The follow-up time started at time of BC diagnosis. It ended when the BC cases deceased, were lost to follow-up or administratively censored by 31^st^ December 2015. Our primary outcome was time to death from lung cancer, as defined by ICD-10 codes C33-34. For BC staging, we used the tumor–node–metastasis (TNM) cancer staging system using the 6^th^ edition BC adjusted stage maintained by the adjusted American Joint Committee on Cancer (22). In order to provide a structured and holistic approach, potential prognostic factors were sub-divided into downstream and upstream factors. In this study, the downstream factors are the clinico-pathological variables (histopathological BC tumor grade and immunohistochemical hormone receptor) while the upstream factors refer to the socio-demographic variables (age at diagnosis, ethnicity, and marital status). Histopathological BC tumor grades are classified as “low grade (well differentiated)”, “moderate grade (moderately differentiated)”, and “high grade (undifferentiated or poorly differentiated)” (23). The values of the variable hormone receptor status were defined based on the different possible combinations of estrogen and progesterone receptors status.

### Statistical Analysis

Since lung cancer incidence and mortality may be age and period dependent (24), estimates of lung cancer attributed mortality between the BC population and the US female general population need to be based on age and calendar period-standardized estimates. In this study, standardized mortality ratios (SMRs) and conditional SMRs (cSMRs) were used to compare lung cancer and all-causes mortality among the BC population with the US female general population. Then, the cSMRs were calculated for BC cases diagnosed between 2000 and 2003, and for the calendar years 2013–2015 on the condition that each BC case achieved a minimum of 10 years survival. In addition, we studied varying time intervals after radiation to investigate how the mortality evolves over time (2005–2006, 2007-2009, and 2010–2012).

In longitudinal medical data, competing risks occur commonly (25). However, the classical survival methods fail to account for possible deaths from competing causes that exhaustively preclude the likelihood of the main cause (26). Competing risk models, like the Fine and Gray model (FGM), are the appropriate statistical approaches for such conditions (27). In contrast to the commonly used FGM, flexible parametric modelling (FPM) allows simultaneous modelling of multiple cause-specific cumulative incidence functions (CIFs) and the effect of potential prognostic factors including time-dependent factors on survival (28, 29). The probability of death from lung cancer and the competing risks (breast cancer and all other causes) was quantified using the cause-specific CIFs (25). This probability was also stratified by the patients’ BC tumor grades to further explore how the different tumor grades impact mortality from the primary outcome taking the effects of the two competing CoD groups into account. Variance inflation factor (VIF) was used to assess multicollinearity at VIF value of ≥ 10. Our assessment showed that the variables BC stage and tumor grade were multicollinear, and thus, the variable BC stage was dropped from the competing risk model. Then, the adjusted effects of the upstream and downstream prognostic factors of mortality from lung cancer, and the competing causes, were modelled using multivariable FPM at P < 0.05. Here, hazard ratios (HR) were estimated with 95% confidence intervals. Stata 14.2 was used to perform the statistical analyses (StataCorp, LP, College Station, TX).

To assess if lung cancer is more frequently observed as a CoD in the high-risk BC cases (the group with metastatic BC) when compared to the general population, a separate analysis was performed (**Supplementary Table 1**). This analysis helps to assess if deaths caused by lung metastases of BC may be misclassified as caused by lung cancer. Here, metastatic cases are treated as a surrogate for early stage BC cases that develop metastases subsequently, and are subject to misclassification of the CoD. If BC patients with thoracic metastases are misclassified as deaths from lung cancer rather than BC, we would find a higher lung cancer related SMRs compared to the general population. However, one would expect a lower lung cancer SMR estimates due to the competing risk of death from BC, which is far more likely in this high-risk group.

We performed another sensitivity analysis in order to estimate the amount of misclassification necessary to negate the observed mortality risk from lung cancer in relation to the general population. Here we estimate the proportion of misclassification required to shift the lower limit of the confidence interval to a value above one. Based on a study by Goldoni et al. (30), we conservatively presumed a maximum misclassification of 10% (maximum proportion reported: 6.5%). We computed the misclassification rate needed according to the following formula

$$p_{mc}=\frac{\frac{n_{lc}}{cSMR}-n_{lc}}{n_{bc}}$$

In this formula, term $\frac{n_{lc}}{cSMR}-n_{lc}$ refers to the number of additional lung cancer deaths needed to negate the effect. Set in relation to the number of observed deaths from BC, we can compute the necessary misclassification rate. In a more realistic approach, we can correct the misclassification rate *p_mc_* to account for the actual proportion of deaths from lung cancer in the population. Here the corrected rate $p{'}_{mc}$ computes as follows

$$p{'}_{mc}=\frac{p_{mc}}{n_{lc}/n_{bc\;}}$$

## Results

### Characteristics by Treatment History and Causes of Death

**Table 1** provides characteristics of 522,982 surgically treated BC cases with respect to the upstream and downstream factors. The majority (60.5%) of the BC cases were treated either by radio-and/or chemotherapy. Of the 316,336 cases treated by radio- and/or chemotherapy, more than four-fifths (81.5%), had undergone lumpectomy. The median follow-up for the treated cases was 6.4 years (interquartile range, 3.0–10.3 years). In comparison to those who received either treatment, those who did not were more likely to be older (**Table 1**).

The proportion of radio‐ and/or chemotherapy treated cases was above the average for estrogen receptor negative (70.8%), progesterone receptor negative (66.4%), and HER2 positive (76%). However, it was lower for the widowed (44.8%), deceased (44.5%), and oldest BC cases (29.8%). About one-fifth were deceased by the end of 2015. The overall mortality was higher (55.5%) for the BC cases with missing information on treatment compared to their counterparts. Of the total 96,316 deceased cases, 4,503 (4.7%), 23,338 (24.2%), and 68,475 (71.1%) were due to lung cancer, BC, and all-other CoD, respectively (**Table 1**).

### Cumulative Mortality Incidence

**Figure 1:**
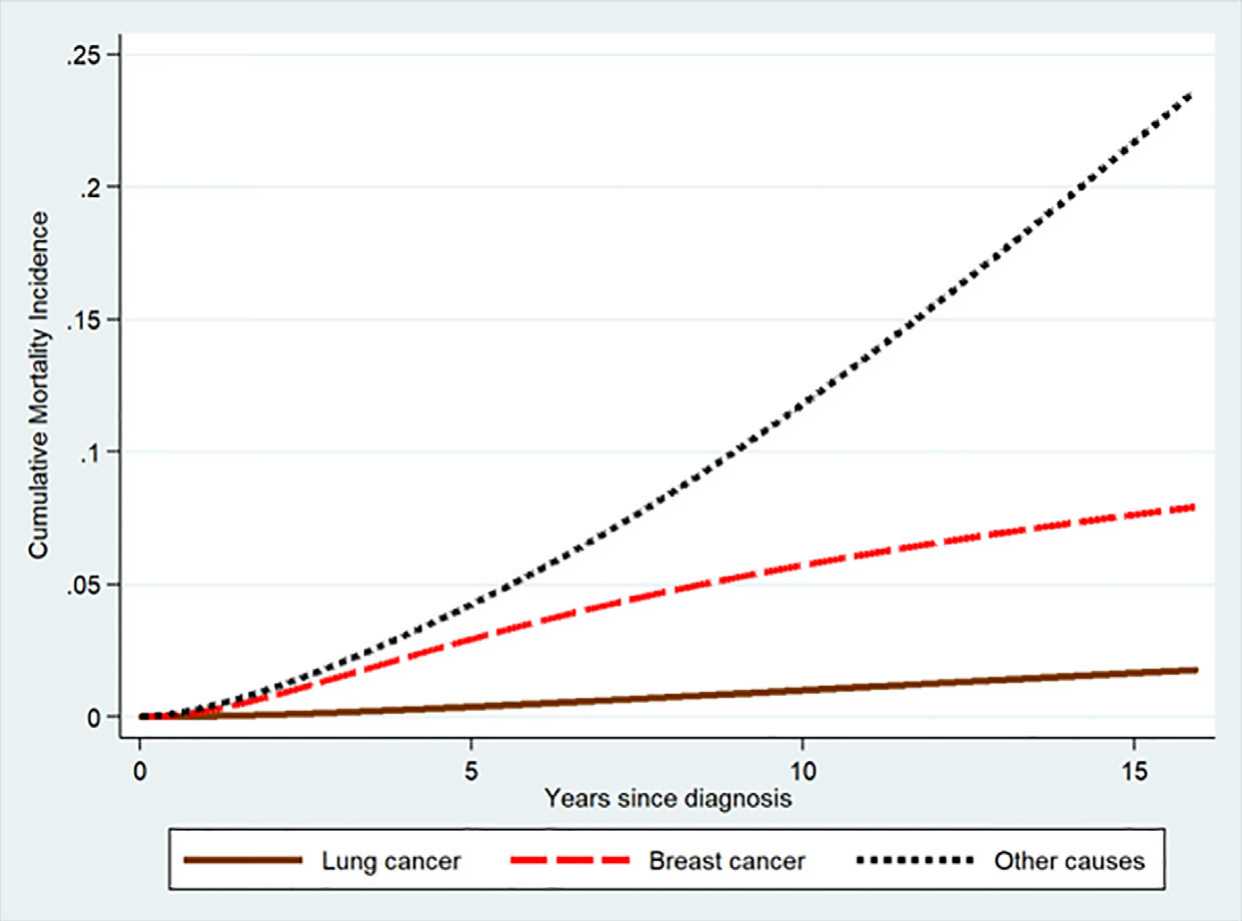
Cause specific-cumulative mortality for breast cancer, lung cancer, and all-other causes among treated early stage BC cases.

In our data (n = 316,336 observations), 2,276 (0.7%) cases deceased from lung cancer, 12,894 (4.1%) from BC, and 27,729 (8.8%) from all-other CoD. **Figure 1** summarizes the cause specific-cumulative mortality incidence (lung cancer, BC, and other causes) for 316,336 BC cases who received radio-and/or chemotherapy. The cumulative mortality caused by lung cancer was the lowest, while the cumulative mortality attributed to all-other CoD was the highest among the three.

**Figure 2:**
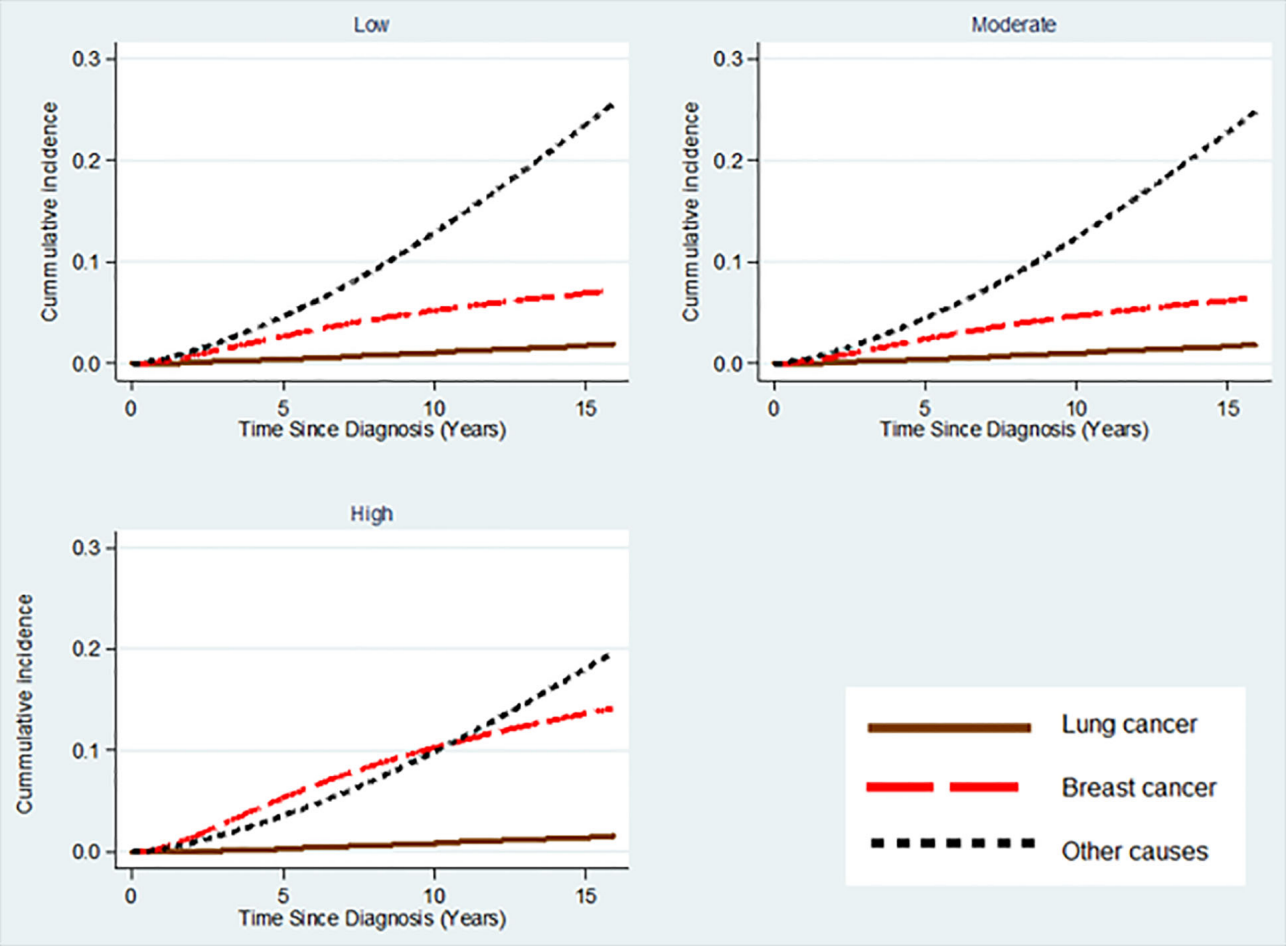
Cumulative mortality of lung cancer, breast cancer, and all-other CoD stratified by histopathological grading of BC tumor among treated early stage breast cancer cases.

**Figure 2** illustrates that the cumulative proportion of BC cases who died by any of the three causes (lung cancer, BC, and all-other CoD) remained below 25% during the follow-up of about 16 years. For low and moderate BC tumor grades, the cumulative mortality of all-other CoD was higher compared to the one for BC. However, for cases with high BC tumor grade, the cumulative mortality caused by BC exceeded that of all-other CoD until about the 10^th^ year post BC diagnosis (**Figure 2**).

### Standardized Mortality Ratios and Conditional Standardized Mortality Ratios

**Table 2** shows the overall standardized mortality ratios (SMRs) for BC cases who were diagnosed and treated between 2000 and 2015. Compared with the lung cancer mortality in the general population, those patients with early stage BC who received only radiotherapy, and radio-and chemotherapy had 24% (95% CI: 28%–20%), and 17% (95% CI: 25%–8%) lower mortality from lung cancer, respectively. However, lung cancer mortality among BC cases treated with chemotherapy (SMR = 0.91; 95% CI: 0.81–1.01) was comparable to the general population.

**Table 2:** Overall standardized mortality ratio (SMR) of lung cancer and all-causes mortality for treated early stage breast cancer cases as compared to the United States female general population, 2000–2015, United States.

Attributes	Categories	Only radiotherapy		Only chemotherapy		Radiotherapy and chemotherapy	
		SMR lung cancer (95% CI)	SMR all causes (95% CI)	SMR lung cancer (95% CI)	SMR all causes (95% CI)	SMR lung cancer (95% CI)	SMR all causes (95% CI)
Age	<55	0.38 (0.23–0.63)	1.32 (1.23–1.42)	1.10 (0.78–1.54)	4.35 (4.16–4.55)	0.98 (0.71–1.35)	3.72 (3.57–3.89)
	55–59	0.66 (0.50–0.85)	1.02 (0.95–1.10)	0.87 (0.61–1.24)	2.64 (2.48–2.82)	0.78 (0.57–1.08)	2.06 (1.93–2.19)
	60–64	0.66 (0.55–0.80)	0.85 (0.81– 0.90)	0.89 (0.67–1.18)	2.26 (2.13–2.39)	0.59 (0.44–0.80)	1.64 (1.54–1.74)
	65–69	0.70 (0.61–0.80)	0.84 (0.80–0.88)	0.77 (0.59–1.00)	1.79 (1.69–1.90)	0.78 (0.62–0.98)	1.32 (1.25–1.40)
	70–74	0.73 (0.65–0.82)	0.84 (0.81–0.87)	0.94 (0.74–1.20)	1.62 (1.52–1.72)	0.77 (0.60–0.98)	1.13 (1.06–1.21)
	75–79	0.82 (0.74–0.91)	0.82 (0.79–0.84)	0.97 (0.73–1.29)	1.33 (1.24–1.42)	1.11 (0.86–1.42)	1.12 (1.04–1.20)
	80–84	0.86 (0.77–0.96)	0.80 (0.78–0.82)	0.92 (0.61–1.37)	1.24 (1.15–1.35)	1.16 (0.82–1.64)	0.91 (0.83–1.00)
Ethnicity	White	0.77 (0.73–0.81)	0.71 (0.69–0.73)	0.98 (0.87–1.11)	1.98 (1.93–2.03)	0.88 (0.78–0.98)	1.52 (1.49–1.56)
	Black	0.85 (0.69–1.05)	0.82 (0.80–0.83)	0.75 (0.51–1.11)	3.22 (3.04–3.42)	0.93 (0.67–1.28)	2.69 (2.54–2.85)
	Others	0.52 (0.40–0.67)	1.23 (1.19–1.27)	0.34 (0.18–0.63)	1.61 (1.47–1.76)	0.17 (0.07–0.40)	1.57 (1.44–1.71)
Marital status	Married	0.63 (0.58–0.68)	0.74 (0.73–0.76)	0.77 (0.66–0.90)	1.92 (1.86–1.98)	0.77 (0.67–0.88)	1.48 (1.43–1.52)
	Single	0.77 (0.64–0.93)	1.07 (1.02–1.12)	1.10 (0.80–1.50)	2.76 (2.60–2.93)	0.78 (0.55–1.09)	2.46 (2.32–2.61)
	Divorced	0.98 (0.84–1.13)	1.08 (1.03–1.12)	1.11 (0.82–1.50)	2.47 (2.32–2.63)	0.88 (0.65–1.19)	1.96 (1.84–2.08)
	Widowed	1.02 (0.92–1.13)	0.89 (0.87–0.92)	1.19 (0.91–1.56)	1.81 (1.69–1.93)	1.11 (0.84–1.46)	1.44 (1.34–1.55)
	Separated & others	0.77 (0.58–1.01)	0.93 (0.87–1.00)	0.93 (0.56–1.55)	2.36 (2.14–2.60)	1.14 (0.73–1.79)	1.74 (1.55–1.94)
Stage	I	0.74 (0.70–0.78)	0.79 (0.77–0.80)	0.90 (0.78–1.05)	1.70 (1.64 –1.76)	0.85 (0.74–0.97)	1.31 (1.27–1.35)
	II	0.94 (0.81–1.09)	1.28 (1.24–1.33)	0.91 (0.77–1.07)	2.52 (2.45–2.60)	0.80 (0.67–0.96)	2.18 (2.11–2.25)
HER2	Positive	0.63 (0.30–1.31)	0.80 (0.67–0.97)	0.23 (0.09–0.61)	1.38 (1.22–1.55)	0.30 (0.13–0.72)	0.91 (0.78–1.06)
	Negative	0.47 (0.39–0.56)	0.56 (0.53–0.58)	0.82 (0.53–1.29)	1.96 (1.79–2.14)	0.42 (0.23–0.76)	1.22 (1.10–1.36)
	Bordeline/ unknown	0.45 (0.22–0.95)	0.71 (0.60–0.85)	1.46 (0.55–3.90)	2.47 (1.96–3.10)	0.46 (0.06–3.26)	1.51 (1.08–2.10)
ER	Positive	0.70 (0.66–0.75)	0.79 (0.78–0.80)	0.84 (0.73–0.98)	1.78 (1.73–1.84)	0.78 (0.68–0.90)	1.38 (1.33–1.42)
	Negative	1.25 (1.07–1.47)	1.33 (1.27–1.39)	1.03 (0.86–1.24)	2.48 (2.40–2.57)	0.89 (0.74–1.06)	2.05 (1.98–2.12)
	Bordeline/ Unknown	0.97 (0.81–1.17)	0.98 (0.93–1.03)	0.85 (0.55–1.31)	2.57 (2.38–2.77)	1.07 (0.71–1.61)	2.10 (1.92–2.29)
PR	Positive	0.70 (0.65–0.74)	0.78 (0.76–0.79)	0.84 (0.71–1.00)	1.76 (1.70–1.82)	0.73 (0.63–0.86)	1.35 (1.30–1.40)
	Negative	0.94 (0.84–1.05)	1.05 (1.02–1.08)	0.98 (0.83–1.15)	2.35 (2.27–2.42)	0.91 (0.78–1.06)	1.91 (1.85–1.97)
	Bordeline/ Unknown	0.91 (0.77–1.08)	0.94 (0.90–0.99)	0.88 (0.60–1.28)	2.35 (2.19–2.52)	1.04 (0.72–1.51)	1.96 (1.81–2.13)
Laterality	Right origin	0.74 (0.68–0.79)	0.85 (0.83–0.87)	0.87 (0.74–1.02)	2.08 (2.01–2.15)	0.87 (0.76–1.01)	1.60 (1.54–1.65)
	Left origin	0.78 (0.73–0.84)	0.83 (0.82–0.85)	0.94 (0.81–1.10)	2.07 (2.00–2.13)	0.79 (0.68–0.92)	1.68 (1.63–1.73)
Overall		0.76 (0.72–0.80)	0.84 (0.83–0.85)	0.91 (0.81–1.01)	2.07 (2.03–2.12)	0.83 (0.75–0.92)	1.64 (1.60–1.68)

HER2, Human epidermal growth factor receptor 2; ER, Estrogen receptor; PR, Progesterone receptor.

Early stage BC cases who received only radiotherapy had 16% (95%CI: 17%–15%) lower mortality from all-CoD compared to the general population. In contrast, BC cases that were treated by radio-and chemotherapy and only chemotherapy had 1.64 (SMR = 1.64; 95% CI: 1.60–1.68), and 2.07 times (SMR = 2.07; 95% CI: 2. 03–2.12) higher mortality from all-CoD compared to the general female population (**Table 2**).

Lung cancer and all-causes mortality, quantified by the overall and factor-specific cSMRs throughout 2013 and 2015, for treated early stage BC cases who were diagnosed between 2000 and 2003 and thus were observed at least for 10 years, as compared to the US general female population are presented in **Table 3**. BC cases who received only radiotherapy (cSMR = 0.93; 95%CI: 0.77–1.13), only chemotherapy (cSMR = 0.91; 95%CI: 0.62–1.33), and radio-and chemotherapy (cSMR = 1.04; 95%CI: 0.77–1.39) had no increased lung cancer mortality relative to the general population. There was evidence of higher all-cause mortality in BC cases who received only chemotherapy (cSMR = 1.32; 95%CI: 1.20–1.45), and radio-and chemotherapy (cSMR = 1.13; 95%CI: 1.04–1.23) in comparison to the general population. In contrast, all-cause mortality was lower (cSMR = 0.87; 95%CI: 0.83–0.92) for those who received only radiotherapy (**Table 3**).

**Table 3:** Overall and factors-specific conditional standardized mortality ratios (cSMRs and 95% CI) for the years 2013–2015 among treated early stage breast cancer cases who were diagnosed between 2000 and 2003 compared with the United States female general population.

Attributes	Categories	Only radiotherapy		Only chemotherapy		Radiotherapy and chemotherapy	
		cSMR lung cancer (95% CI)	cSMR all-causes (95% CI)	cSMR lung cancer (95% CI)	cSMR all-causes (95% CI)	cSMR lung cancer (95% CI)	cSMR all-causes (95% CI)
Age	< 55	1.18 (0.17–8.40)	1.07 (0.61–1.88)	–	2.05 (1.45–2.90)	–	2.56 (1.97–3.32)
	55–59	0.72 (0.18–2.89)	1.31 (0.96–1.80)	0.83 (0.21–3.33)	1.95 (1.47–2.58)	0.57 (0.14–2.29)	1.58 (1.22–2.05)
	60–64	0.87 (0.39–1.93)	0.87 (0.68–1.12)	0.52 (0.13–2.06)	1.53 (1.19–1.97)	0.34 (0.08–1.34)	1.14 (0.90–1.44)
	65–69	0.49 (0.24–0.94)	0.81 (0.68–0.97)	0.50 (0.16–1.54)	1.34 (1.07–1.68)	1.16 (0.64–2.10)	0.97 (0.78–1.20)
	70–74	0.98 (0.66–1.43)	0.85 (0.74–0.97)	0.73 (0.30–1.74)	1.22 (0.98–1.51)	0.69 (0.33–1.44)	1.01 (0.83–1.23)
	75–79	1.05 (0.75–1.48)	0.89 (0.80–0.99)	1.74 (0.94–3.23)	1.17 (0.94–1.45)	1.86 (1.12–3.09)	1.08 (0.89–1.30)
	80–84	1.03 (0.73–1.45)	0.86 (0.79–0.94)	1.37 (0.57–3.30)	1.05 (0.83–1.32)	1.67 (0.83–3.34)	0.94 (0.75–1.16)
Stage	I	0.88 (0.72–1.08)	0.85 (0.81–0.90)	0.69 (0.39–1.22)	1.25 (1.10– 1.42)	1.02 (0.71–1.46)	1.01 (0.91–1.13)
	II	1.64 (0.95–2.83)	1.13 (0.95–1.36)	1.21 (0.73–2.01)	1.42 (1.24–1.63)	1.07 (0.65–1.74)	1.36 (1.19–1.54)
Tumor grade	Low	1.16 (0.87–1.53)	0.85 (0.78–0.93)	1.15 (0.43–3.05)	1.20 (0.90–1.59)	1.12 (0.51–2.50)	1.06 (0.83–1.36)
	Moderate	0.83 (0.61–1.13)	0.82 (0.75–0.89)	1.13 (0.64–1.99)	1.43 (1.23–1.66)	1.08 (0.67–1.73)	1.17 (1.02–1.34)
	High	0.83 (0.49–1.39)	1.03 (0.90–1.17)	0.67 (0.35–1.29)	1.30 (1.13– 1.49)	0.86 (0.53–1.38)	1.09 (0.96–1.23)
ER	Positive	0.95 (0.78–1.17)	0.86 (0.81–0.91)	1.04 (0.66–1.65)	1.36 (1.20–1.53)	1.21 (0.85–1.72)	1.20 (1.08–1.34)
	Negative	0.85 (0.41–1.78)	1.04 (0.86–1.25)	0.78 (0.37–1.63)	1.17 (0.98–1.39)	0.70 (0.38–1.30)	1.00 (0.86–1.17)
PR	Positive	0.91 (0.72–1.14)	0.84 (0.79–0.90)	0.93 (0.54–1.60)	1.32 (1.15–1.51)	1.16 (0.79–1.72)	1.21 (1.08–1.36)
	Negative	0.99 (0.64–1.51)	0.98 (0.87–1.10)	0.88 (0.47–1.64)	1.27 (1.09–1.47)	0.85 (0.51–1.40)	1.03 (0.90–1.18)
Laterality	Right origin	0.85 (0.65–1.13)	0.91 (0.84–0.98)	0.84 (0.48–1.48)	1.37 (1.20–1.56)	1.01 (0.66–1.53)	1.11 (0.98–1.25)
	Left origin	1.01 (0.79–1.30)	0.84 (0.78–0.91)	0.98 (0.59–1.62)	1.28 (1.12–1.46)	1.07 (0.71–1.60)	1.16 (1.03–1.30)
Overall		0.93 (0.77–1.13)	0.87 (0.83–0.92)	0.91 (0.62–1.33)	1.32 (1.20–1.45)	1.04 (0.77–1.39)	1.13 (1.04–1.23)

ER, Estrogen receptor; PR, Progesterone receptor.

The cSMRs were similar for shorter lack intervals since diagnosis indicating a constant effect over time (**Table 4**).

**Table 4:** Conditional SMRs (cSMRs and 95% CI) of lung cancer and all-causes attributed mortality for early stage BC cases diagnosed between 2000 and 2003 by calendar periods and treatment groups compared to the general United States female population.

Calendar period	Only radiotherapy		Only chemotherapy		Radiotherapy and chemotherapy	
	cSMR lung cancer (95% CI)	cSMR all-causes (95% CI)	cSMR lung cancer (95% CI)	cSMR all-causes (95% CI)	cSMR lung cancer (95% CI)	cSMR all-causes (95% CI)
2005–2006	0.69 (0.55–0.85)	0.96 (0.91–1.02)	1.22 (0.81–1.81)	2.88 (2.65–3.12)	0.86 (0.57–1.31)	2.30 (2.13–2.50)
2007–2009	1.02 (0.88–1.19)	1.05 (1.01–1.10)	1.20 (0.87–1.65)	2.28 (2.12–2.45)	1.13 (0.85–1.51)	1.89 (1.77–2.03)
2010–2012	1.07 (0.92–1.25)	1.03 (0.98–1.07)	0.70 (0.46–1.07)	1.82 (1.68–1.97)	0.82 (0.59–1.14)	1.54 (1.44–1.66)

### Sensitivity Analysis

Results from the sub-group analysis of the metastatic cases revealed higher mortality from both lung cancer and all CoD for all the treatment schemes relative to the general population (**Supplementary Table 1**). However, in the sensitivity analysis based on the sub-group with only radiotherapy, we found that the required proportion of misclassified cases would exceed 10.8%. This is above the rate estimated in a previous study be Goldoni et al. (6.5%). Thus, misclassification is unlikely to have the potential to explain our findings of a comparable lung cancer mortality. Considering the corrected misclassification rate, the rate of misclassification would need to exceed 100%.

### Downstream and Upstream Factors Associated With Lung Cancer Attributed Mortality

Controlling the effects of the fitted independent variables, negative estrogen and progesterone receptor status (HR = 1.38; 95% CI: 1.21–1.57) was a strong downstream factor related to increased lung cancer attributed mortality. The upstream factors associated with increased mortality were older age and unmarried family status (**Table 5**). Compared to those younger than 50 years, those who were in the age groups of 50–59 and 60–69 years had more than three-fold (HR_50-59_ = 3.41; 95% CI: 2.72–4.28), and seven-fold (HR_60-69_ = 7.33; 95% CI: 5.90–9.10) hazards of lung cancer-attributed mortality. This effect continued in older age groups. Comparing with BC cases who were married, those who were widowed, divorced, single or separate marital status had 76% (95% CI: 58%–97%), 45% (95% CI: 26%–66%) and 25% (95% CI: 10%–43%) higher hazards of mortality from lung cancer, respectively (**Table 5**). When compared to BC cases from white ethnic background, lung cancer attributed mortality was comparably lower for American Indian/Alaska Native and Asian/Pacific Islander ethnicities (HR = 0.51; 95% CI: 0.40–0.64). Unlike their strong associations with elevated mortality from the competing causes, we found no increased lung cancer-ascribed mortality for high BC tumor grade (HR = 1.05; 95% CI: 0.93–1.19) and black ethnicity (HR = 0.92; 95% CI: 0.78–1.09) (**Table 5**).

**Table 5:** Downstream and upstream factors identified using multivariable flexible parametric hazards model, hazard ratios (HRs) with 95% confidence intervals (CIs), for lung cancer-specific, breast cancer and all-other causes of death.

Characteristics	Hazard ratios and 95% CI		
	Lung cancer	Breast cancer	All-other causes
**Age at diagnosis**			
<50	1.00	1.00	1.00
50–59	3.41 (2.72–4.28)	0.92 (0.88–0.97)	1.92 (1.80–2.05)
60–69	7.33 (5.90–9.10)	0.97 (0.92–1.02)	4.56 (4.29–4.84)
70–79	10.53 (8.44–13.13)	1.26 (1.19–1.34)	12.07 (11.37–12.82)
≥ 80	10.42 (8.08–13.45)	1.96 (1.81–2.12)	29.07 (27.29–30.97)
**Ethnicity**			
White	1.00	1.00	1.00
Black	0.92 (0.78–1.09)	1.38 (1.31–1.46)	1.26 (1.20–1.32)
Others	0.51 (0.40–0.64)	0.87 (0.81–0.94)	0.75 (0.71–0.79)
**Marital status**			
Married	1.00	1.00	1.00
Single/separated/others	1.25 (1.10–1.43)	1.17 (1.11–1.23)	1.34 (1.28–1.39)
Divorced	1.45 (1.26–1.66)	1.19 (1.12–1.26)	1.38 (1.33–1.44)
Widowed	1.76 (1.58–1.97)	1.27 (1.20–1.35)	1.40 (1.36–1.44)
**Histopathological grading of breast cancer tumor**			
Low grade	1.00	1.00	1.00
Moderate	1.01 (0.91–1.12)	2.29 (2.15–2.45)	1.04 (1.01–1.07)
High grade	1.05 (0.93–1.19)	4.21 (3.93–4.51)	1.14 (1.10–1.18)
**Estrogen receptor (ER) and progestrone receptor (PR)**			
ER+ and PR+	1.00	1.00	1.00
ER- and PR-	1.38 (1.21–1.57)	1.89 (1.80–1.98)	1.05 (1.01–1.09)
ER+ and PR-	1.12 (0.98–1.27)	1.58 (1.49–1.67)	1.00 (0.97–1.04)
ER- and PR+	1.17 (0.78–1.78)	1.77 (1.57–2.01)	0.97 (0.86–1.11)
Other combinations	1.20 (1.03–1.40)	1.59 (1.49–1.70)	1.05 (1.00–1.09)

## Discussion

Our study provided no evidence for a difference in lung cancer attributed mortality between treated early stage BC cases, as quantified by cSMRs, and the general US female population. We also identified prognostic factors associated with lung cancer mortality and competing causes of death among treated early stage BC cases.

As demonstrated by Taylor et al. the excess risk would have different implications for smokers and non-smokers (15). Even if modern regimes are still likely to cause various cancers including lung cancer, quitting smoking might decrease the overall cancer mortality of BC patients to the same or even lower level of the general population.

Interestingly, apart from the first 5 years in patients who received radiation only, we could not observe a further reduction of lung cancer mortality, which one could expect due to intensive screening and the encouragement of patients to change their life style. This conversely could indicate that sequential factors such as clinical follow-up and/or a more favorable life style might compensate for the possibly adverse effects of radiotherapy. The estimates are not precise enough to provide a final answer and the observation period of 10–15 years might be too short to reveal an elevated risk.

However, there have been improvements in treatment planning, radiation-dose optimization, and sparing of surrounding tissues with the recent radiotherapy techniques in most recent times (14, 31). Due to such continual improvements, the risks of radiotherapy-induced secondary cancers and related deaths are likely to have decreased (13–15, 32). Nevertheless, studies have shown that irradiation of BC was associated with increased deleterious impacts to the lungs of smokers, including elevated symptomatic treatment-related side effects (15, 33). Therefore, efforts to assess and implement smoking cessation strategies among active smokers of BC patients, along with focused lung cancer screening, should be essential components of the treatment plan before the initiation of radiotherapy (13, 15). The ‘treatment favouring’ cSMR estimates in 2005 to 2006 (**Table 4**) may rather reflect the close oncologic follow-up in the first 5 years after diagnosis making an early detection of lung cancer more likely. Thus, people might avoid deaths from lung cancer by a comprehensive screening related to the sequential radiologic examination of the lung after the initial treatment and, consequently, the diagnosis of lung cancer in an early stage with a favorable prognosis. SMRs for all-cause mortality close to one support this notion as the non-cancer-related mortality remains unaffected by radiologic screening or follow-up. In this study, the conditional SMRs for the all-CoD group was higher for the BC cases treated by chemo-and radiotherapy or only chemotherapy, unlike for those that received only radiotherapy. The main explanation for this is that BC cases which receive chemotherapy are likely to be at advanced stage, as compared to those who receive only radiotherapy.

With the goal of identifying the upstream and downstream factors associated with lung cancer mortality, we performed our analysis only among the treated patients with early stage BC. Triple negative BC cases have higher risk of developing second primary lung cancer and are exposed to a higher risk of lung cancer mortality (12, 34). Previous studies found an elevated risks for lung cancer in estrogen negative (ER-) or progesterone negative (PR-) BC, even before radiotherapy treatment (12, 35). Our finding of an increased hazard of mortality due to lung cancer among BC cases with negative immunohistochemical hormone receptors (ER- and PR) concurs with these findings.

Older cancer patients are more likely to have higher burden of comorbidity, and to suffer from aging-related adverse health conditions (36, 37) and could thus have a worse prognosis (38). This may partly explain the apparently increased mortality, from both lung cancer and the competing causes, for older BC patients observed in this study. In addition, our results showed that unmarried cases were strongly associated with a higher hazard of mortality from lung cancer, BC and all-other CoD. Studies link unmarried patients with undertreatment, and higher cancer-specific and all-cause mortality (39–41). On the other hand, being married was associated with better social support and adherence to cancer treatment (40, 41). The inverse association of being married and mortality could be also due to interacting effect of income status (41). However, it was shown that the protective effect of married status on cancer-specific and all-cause mortality was independent of income status (39, 40). Therefore, the current findings may be highlighting the importance of delivering psychosocial support to BC survivors as part of their care and treatment, especially for those who have less family support. In this study, higher tumor grade and black ethnicity were not associated with increased lung cancer mortality, but with mortality from BC and all-other CoD.

We tried to estimate the degree of misclassification of the CoD from thoracic metastases of BC registered as lung cancer deaths possible to negate observed effects (**Supplementary Table 1**). The SMRs calculated for the high-risk (metastatic) group revealed that lung cancer mortality was higher for the BC cases as compared to the general population. This finding may reflect a scenario of misclassifying BC caused deaths as if they were due to lung cancer, overestimating lung cancer mortality among BC patients. However even if reverse relations hold, considering the results of the sensitivity analysis, misclassification is unlikely to explain our findings. Still, the presumptions used for the sensitivity analysis are very conservative, as misclassification is subject to not only lung and breast cancer but to all-other CoD.

Although our study is based on big, gold standard and representative cancer registry data (18), it has the following limitations. First, the overall utility of the SEER data to reliably identify cancer treatment service was reported to be limited although it was shown to have high positive predictive value of capturing BC treatment (42, 43). Second, the follow-up time of this study, maximum of about 16 years with a median of 6.4 years, is limited. Indeed, evidence from SEER data, based on irradiations prior to 2009, showed increased lung-cancer mortality risk in second and third decades after exposure (14, 16). Another limitation of the SEER database was that it collects no data on smoking status, income, and comorbidities such as chronic obstructive pulmonary diseases and interstitial lung disease; due to this, their possible confounding effect was unaccounted for in this study. Similarly, information on radiotherapy technique, and detailed dosimetric information (including radiation dose leaked into normal lungs from BC irradiation), that may influence likelihood of lung cancer incidence and related mortality, was not documented. In addition, data regarding types of chemotherapy received by the patients, and clinically important molecular profiling of lung cancer data on epidermal growth factor receptor (EGFR), anaplastic lymphoma kinase (AKA), and programmed death-1 (PD-1) were also not captured. Finally, the findings of this study apply only to early stage BC cases, excluding the triple negative cases.

## Conclusion

In summary, this study has shown that early stage BC cases (T1-T3N_0_), who were under different treatment modalities, had no higher risk of mortality from lung cancer relative to their background population. On the ground of the current findings, lung cancer mortality should not be a major concern in the treatment decision of early stage BC cases. Negative immunohistochemical hormone receptor was the strong downstream factor whereas patients with older age, and unmarried relationships were the strong upstream factors for lung cancer mortality. Targeting these factors could benefit early stage BC cases in terms of reducing mortality from lung cancer as well as from the competing risks. It is important to note that our findings apply only to treated early stage BC cases, and they are by no means inferable to all other BC cases including the triple negative cases.

## Data Availability Statement

The original contributions presented in the study are publicly available. This data can be found here: SEER database, https://seer.cancer.gov/data/sample-dua.html.

## Ethics Statement

Ethical review and approval was not required for the study on human participants in accordance with the local legislation and institutional requirements. Written informed consent for participation was not required for this study in accordance with the national legislation and the institutional requirements.

## Author Contributions

SA: wrote parts of the manuscript and performed the analyses. RM: wrote parts of the manuscript, contributed to the statistical methods, read the manuscript critically. EK: wrote parts of the manuscript, read the manuscript critically, clinical review. LE: wrote parts of the manuscript, read the manuscript critically. AB: contributed to the statistical methods, read the manuscript critically. CO: read the manuscript critically, contributed to the methods. AG: wrote parts of the manuscript, read the manuscript critically, clinical review. DV: wrote parts of the manuscript, read the manuscript critically, clinical review. DM: had the initial idea, contributed to the statistical methods, wrote parts of the manuscript, read the manuscript critically, clinical review. All authors contributed to the article and approved the submitted version.

## Funding

We acknowledge the financial support within the funding programme Open Access Publishing by the German Research Foundation (DFG). The funders had no role in study design, data collection and analysis, decision to publish, or preparation of the manuscript.

## Conflict of Interest

The authors declare that the research was conducted in the absence of any commercial or financial relationships that could be construed as a potential conflict of interest.

## References

[1] Ghoncheh M Pournamdar Z Salehiniya H. Incidence and Mortality and Epidemiology of Breast Cancer in the World. Asian Pac J Cancer Prev (2016) 17(3):43–6. 10.7314/APJCP.2016.17.S3.43 27165206

[2] Institute for Health Metrics and Evaluation. Global Burden of Disease Study 2017 Results. Glob Burd Dis Collab Netw (2017). Available at: https://vizhub.healthdata.org/gbd-compare/ (Accessed June 15, 2020).

[3] Miller KD Nogueira L Mariotto AB Rowland JH Yabroff KR Alfano CM. Cancer treatment and survivorship statistics, 2019. CA Cancer J Clin (2019) 69(5):363–85. 10.3322/caac.21565 31184787

[4] Siegel RL Miller KD Jemal A. Cancer statistics, 2019. CA Cancer J Clin (2019) 69(1):7–34. 10.3322/caac.21551 30620402

[5] Tao ZQ Shi A Lu C Song T Zhang Z Zhao J. Breast Cancer: Epidemiology and Etiology. Cell Biochem Biophys (2015) 72(2):333–8. 10.1007/s12013-014-0459-6 25543329

[6] Shah R Rosso K David Nathanson S. Pathogenesis, prevention, diagnosis and treatment of breast cancer. World J Clin Oncol (2014) 5(3):283–98. 10.5306/wjco.v5.i3.283 PMC4127601 25114845

[7] Kesson EM Allardice GM George WD Burns HJG Morrison DS. Effects of multidisciplinary team working on breast cancer survival: Retrospective, comparative, interventional cohort study of 13 722 women. BMJ (2012) 344:e2718. 10.1136/bmj.e2718 22539013 PMC3339875

[8] Byers T Wender RC Jemal A Baskies AM Ward EE Brawley OW. The American Cancer Society challenge goal to reduce US cancer mortality by 50% between 1990 and 2015: Results and reflections. CA Cancer J Clin (2016) 66(5):359–69. 10.3322/caac.21348 27175568

[9] Moulder S Hortobagyi GN. Advances in the treatment of breast cancer. Clin Pharmacol Ther (2008) 83(1):26–36. 10.1038/sj.clpt.6100449 18091763

[10] Miller KD Siegel RL Lin CC Mariotto AB Kramer JL Rowland JH. Cancer treatment and survivorship statistics, 2016. CA Cancer J Clin (2016) 66(4):271–89. 10.3322/caac.21349 27253694

[11] Coleman MP Forman D Bryant H Butler J Rachet B Maringe C. Cancer survival in Australia, Canada, Denmark, Norway, Sweden, and the UK, 1995-2007 (the international cancer benchmarking partnership): An analysis of population-based cancer registry data. Lancet (2011) 377(9760):127–38. 10.1016/S0140-6736(10)62231-3 PMC3018568 21183212

[12] Liu J Hu Z Feng Y Zeng S Zhong M. Problems to affect long-term survival for breast cancer patients An observational study of subsequent lung/bronchus malignancies. Med (United States) (2018) 97(39):e12603. 10.1097/MD.0000000000012603 PMC6181575 30278574

[13] Lorigan P Califano R Faivre-Finn C Howell A Thatcher N. Lung cancer after treatment for breast cancer. Lancet Oncol (2010) 11(12):1184–92. 10.1016/S1470-2045(10)70056-5 20541465

[14] Darby SC McGale P Taylor CW Peto R. Long-term mortality from heart disease and lung cancer after radiotherapy for early breast cancer: Prospective cohort study of about 300 000 women in US SEER cancer registries. Lancet Oncol (2005) 6(8):557–65. 10.1016/S1470-2045(05)70251-5 16054566

[15] Taylor C Correa C Duane FK Aznar MC Anderson SJ Bergh J. Estimating the Risks of Breast Cancer Radiotherapy: Evidence From Modern Radiation Doses to the Lungs and Heart and From Previous Randomized Trials. J Clin Oncol (2017) 35(15):1641–9. 10.1200/JCO.2016.72.0722 PMC5548226 28319436

[16] Henson KE McGale P Taylor C Darby SC. Radiation-related mortality from heart disease and lung cancer more than 20 years after radiotherapy for breast cancer. Br J Cancer (2013) 108(1):179–82. 10.1038/bjc.2012.575 PMC3553540 23257897

[17] Grantzau T Overgaard J. Risk of second non-breast cancer among patients treated with and without postoperative radiotherapy for primary breast cancer: A systematic review and meta-analysis of population-based studies including 522,739 patients. Radiother Oncol (2016) 121(3):402–13. 10.1016/j.radonc.2016.08.017 27639892

[18] Duggan MA Anderson WF Altekruse S Penberthy L Sherman ME. The surveillance, epidemiology, and end results (SEER) program and pathology: Toward strengthening the critical relationship. Am J Surg Pathol (2016) 40(12):e94–e102. 10.1097/PAS.0000000000000749 27740970 PMC5106320

[19] World Health Organization. International Statistical Classification of Diseases and Related Health Problems (International Classification of Diseases) (ICD) 10th Revision. (2010). Available online at: https://icd.who.int/browse10/2010/en (Accessed January 26, 2019).

[20] Center for Disease Control and Prevention. Wide-ranging online data for epidemiologic research (WONDER). CDC (2016). Available online at: http://wonder.cdc.gov/ (Accessed July 8, 2019).

[21] Friede A Rosen DH Reid JA. CDC WONDER: A cooperative processing architecture for public health. J Am Med Inf Assoc (1994) 1(4):303–12. 10.1136/jamia.1994.95236162 PMC116209 7719813

[22] Edge SB Compton CC. The american joint committee on cancer: The 7th edition of the AJCC cancer staging manual and the future of TNM. Ann Surg Oncol (2010) 17(6):1471–4. 10.1245/s10434-010-0985-4 20180029

[23] Jemal A Robbins AS Lin CC Flanders WD DeSantis CE Ward EM. Factors that contributed to black-white disparities in survival among nonelderly women with breast cancer between 2004 and 2013. J Clin Oncol (2018) 36(1):14–24. 10.1200/JCO.2017.73.7932 29035645

[24] Tas F Ciftci R Kilic L Karabulut S. Age is a prognostic factor affecting survival in lung cancer patients. Oncol Lett (2013) 6(5):1507–13. 10.3892/ol.2013.1566 PMC3813578 24179550

[25] Kim HT. Cumulative incidence in competing risks data and competing risks regression analysis. Clin Cancer Res (2007) 13(2 Pt 1):559–65. 10.1158/1078-0432.CCR-06-1210 17255278

[26] Koller MT Raatz H Steyerberg EW Wolbers M. Competing risks and the clinical community: Irrelevance or ignorance? Stat Med (2012) 31(11-12):1089–97. 10.1002/sim.4384 PMC3575691 21953401

[27] Austin PC Fine JP. Practical recommendations for reporting Fine-Gray model analyses for competing risk data. Stat Med (2017) 36(27):4391–400. 10.1002/sim.7501 PMC5698744 28913837

[28] Fine JP Gray RJ. A Proportional Hazards Model for the Subdistribution of a Competing Risk. J Am Stat Assoc (1999) 94(446):496–509. 10.1080/01621459.1999.10474144

[29] Mozumder SI Rutherford M Lambert P. Direct likelihood inference on the cause-specific cumulative incidence function: A flexible parametric regression modelling approach. Stat Med (2018) 37(1):82–97. 10.1002/sim.7498 28971494 PMC6175037

[30] Goldoni CA Bonora K Ciatto S Giovannetti L Patriarca S Sapino A. Misclassification of breast cancer as cause of death in a service screening area. Cancer Causes Control (2009) 20(5):533–8. 10.1007/s10552-008-9261-3 19015942

[31] Saw CB Li S. 3D treatment planning systems. Med Dosim (2018) 43(2):103–5. 10.1016/j.meddos.2018.03.002 29753333

[32] Orecchia R Lucignani G. Early-stage breast cancer: Risk of heart disease and lung cancer. Lancet Oncol (2005) 6(8):539–40. 10.1016/S1470-2045(05)70258-8 16054563

[33] Peppone LJ Mustian KM Morrow GR Dozier AM Ossip DJ Janelsins MC. The Effect of Cigarette Smoking on Cancer Treatment-Related Side Effects. Oncologist (2011) 16(12):1784–92. 10.1634/theoncologist.2011-0169 PMC3248778 22135122

[34] Wang R Yin Z Liu L Gao W Li W Shu Y. Second Primary Lung Cancer After Breast Cancer: A Population-Based Study of 6,269 Women. Front Oncol (2018) 8:427. 10.3389/fonc.2018.00427 30356721 PMC6189405

[35] Schonfeld SJ Curtis RE Anderson WF Berrington De González A. The risk of a second primary lung cancer after a first invasive breast cancer according to estrogen receptor status. Cancer Causes Control (2012) 23(10):1721–8. 10.1007/s10552-012-0054-3 PMC3474934 22918549

[36] Paillaud E Liuu E Laurent M Le Thuaut A Vincent H Raynaud-Simon A. Geriatric syndromes increased the nutritional risk in elderly cancer patients independently from tumoursite and metastatic status. The ELCAPA-05 cohort study. Clin Nutr (2014) 33(2):330–5. 10.1016/j.clnu.2013.05.014 23786899

[37] Gironés R Torregrosa D Díaz-Beveridge R. Comorbidity, disability and geriatric syndromes in elderly breast cancer survivors. Results of a single-center experience. Crit Rev Oncol Hematol (2010) 73(3):236–45. 10.1016/j.critrevonc.2009.08.002 19748793

[38] Patnaik JL Byers T Diguiseppi C Denberg TD Dabelea D. The influence of comorbidities on overall survival among older women diagnosed with breast cancer. J Natl Cancer Inst (2011) 103(14):1101–11. 10.1093/jnci/djr188 PMC3139585 21719777

[39] Hinyard L Wirth LS Clancy JM Schwartz T. The effect of marital status on breast cancer-related outcomes in women under 65: A SEER database analysis. Breast (2017) 32:13–7. 10.1016/j.breast.2016.12.008 28012410

[40] Aizer AA Chen MH McCarthy EP Mendu ML Koo S Wilhite TJ. Marital status and survival in patients with cancer. J Clin Oncol (2013) 31(31):3869–76. 10.1200/JCO.2013.49.6489 PMC4878087 24062405

[41] Martínez ME Unkart JT Tao L Kroenke CH Schwab R Komenaka I. Prognostic significance of marital status in breast cancer survival: A population-based study. PloS One (2017) 12(5):e0175515. 10.1371/journal.pone.0175515 28475579 PMC5419505

[42] Noone AM Lund JL Mariotto A Cronin K McNeel T Deapen D. Comparison of SEER Treatment Data With Medicare Claims. Med Care (2016) 54(9):e55–64. 10.1097/MLR.0000000000000073 PMC4981219 24638121

[43] Jagsi R Abrahamse P Hawley ST Graff JJ Hamilton AS Katz SJ. Underascertainment of radiotherapy receipt in surveillance, epidemiology, and end results registry data. Cancer (2012) 118(2):333–41. 10.1002/cncr.26295 PMC3224683 21717446

