# Supplementary material for: Lung Cancer Attributed Mortality Among 316,336 Early Stage Breast Cancer Cases Treated by Radiotherapy and/or Chemotherapy, 2000–2015: Evidence From the SEER Database
Source: Front Oncol. 2021 Feb 25;10:602397. doi: 10.3389/fonc.2020.602397 (PMC7947230; doi:10.3389/fonc.2020.602397)
Supplement: Supplementary file 1 [file DataSheet_1.pdf]

Supplementary files

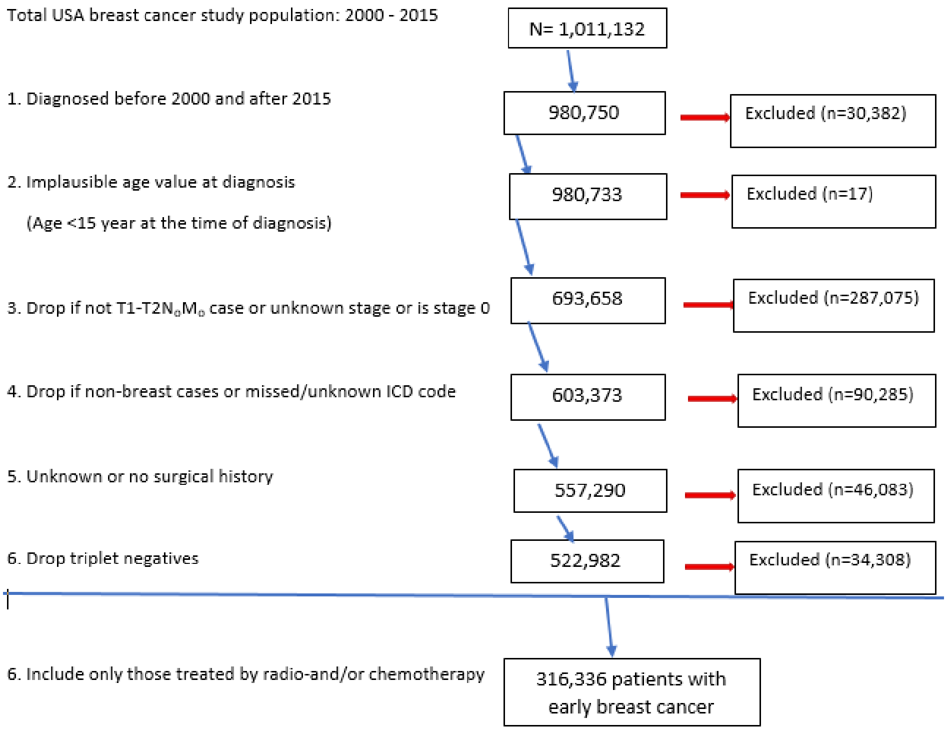

Figure S1. Eligibility criteria for the competing risk model

Table S1. Overall SMRs (and 95% CI) of lung cancer and all-causes attributed mortality by treatment groups for metastasized BC cases, diagnosed between 2000 and 2003, compared to the general US female population

Characteristics	Categories	SMRs by Breast cancer treatment types					
		Only radiotherapy		Only chemotherapy		Radiotherapy and chemotherapy	
		SMRs lung cancer (95% CI)	SMRs all-causes (95% CI)	SMRs lung cancer (95% CI)	SMRs all-causes (95% CI)	SMRs lung cancer (95% CI)	SMRs all-causes (95% CI)
Age	< 55	3.77 (0.53 - 26.7)	66.2 (57.7 - 76.0)	5.12 (2.30 - 11.4)	69.1 (64.9 - 73.5)	8.60 (4.3 - 17.2)	63.6 (59.1 - 68.4)
	55 - 59	5.24 (1.31 - 20.9)	33.1 (27.4 - 40.0)	2.30 (0.74 - 7.12)	30.1 (27.1 - 33.5)	3.06 (0.99 - 9.49)	26.1 (22.8 - 29.8)
	60 - 64	-	21.3 (18.0 - 25.2)	1.76 (0.57 - 5.44)	22.8 (20.5 - 25.5)	3.37 (1.26 - 8.90)	19.1 (16.6 - 22.1)
	65 - 69	2.00 (0.50 - 7.98)	14.8 (12.5 - 17.5)	4.33 (2.16 - 8.65)	15.9 (14.1 - 17.9)	2.33 (0.75 - 7.23)	13.7 (11.7 - 15.9)
	70 - 74	2.68 (0.86 - 8.32)	12.9 (11.1 - 15.1)	2.09 (0.78 - 5.57)	11.7 (10.3 - 13.2)	3.75 (1.21 - 11.6)	11.3 (9.3 - 13.7)
	75 - 79	2.32 (0.75 - 7.18)	8.4 (7.2 - 9.7)	4.90 (2.20 - 10.9)	8.8 (7.5 - 10.2)	1.43 (0.20 - 10.1)	8.0 (6.5 - 9.9)
	80 - 84	5.38 (1.73 - 16.7)	6.21 (5.0 - 7.7)	-	8.4 (6.6 - 10.8)	-	6.7 (4.7 - 9.6)
BC tumor grade	Low	4.06 (0.57 - 28.8)	13.5 (9.7 - 18.8)	-	12.1 (9.5 - 15.3)	-	15.3 (11.6 - 20.3)
	Moderate	2.55 (1.06 - 6.12)	11.6 (10.4 - 13.1)	1.43 (0.54 - 3.82)	18.6 (17.1 - 20.2)	1.46 (0.47 - 4.53)	15.3 (13.8 - 17.1)
	High	2.62 (0.98 - 6.98)	17.9 (16.2 - 19.9)	3.69 (2.19 - 6.24)	26.7 (25.2 - 28.3)	4.11 (2.21 - 7.64)	29.6 (27.6 - 31.8)
ER+	Positive	1.89 (0.90 - 3.97)	13.5 (12.5 - 14.6)	1.64 (0.85 - 3.15)	18.8 (17.7 - 19.9)	2.72 (1.50 - 4.90)	17.8 (16.5 - 19.1)
	Negative	5.93 (1.48 - 23.7)	26.1 (21.7 - 31.3)	4.24 (2.12 - 8.49)	32.9 (30.5 - 35.6)	6.81 (3.25 - 14.3)	37.1 (33.6 - 40.9)
PR+	Positive	1.95 (0.87 - 4.33)	12.4 (11.3 - 14.7)	1.64 (0.78 - 3.43)	18.1 (17.0 - 19.4)	2.86 (1.49 - 5.50)	17.1 (15.8 - 18.6)
	Negative	2.16 (0.54 - 8.65)	20.6 (18.1 - 23.5)	2.82 (1.41 - 5.64)	28.5 (26.7 - 30.5)	4.38 (2.19 - 8.76)	29.7 (27.3 - 32.2)
Laterality	Right origin	1.53 (0.58 - 4.10)	14.2 (12.9 - 15.5)	2.23 (1.16 - 4.29)	25.1 (23.6 - 26.6)	2.83 (1.42 - 5.66)	22.7 (21.1 - 24.5)
	Left origin	4.00 (2.15 - 7.13)	14.8 (13.5 - 16.2)	3.27 (2.00 - 5.33)	21.8 (20.6 - 23.1)	2.66 (1.33 - 5.31)	22.9 (21.3 - 24.6)
Overall		2.59 (1.54 - 4.38)	14.8 (13.9 - 15.7)	3.15 (2.20 - 4.51)	23.1 (22.2 - 24.1)	3.62 (2.38 - 5.49)	22.9 (21.8 - 24.1)
